# The Role of Cyclic Adenosine Monophosphate (cAMP) in Modulating Glucocorticoid Receptor Signaling and Its Implications on Glucocorticoid-Related Collagen Loss

**DOI:** 10.3390/ijms241210180

**Published:** 2023-06-15

**Authors:** Wesuk Kang, Dabin Choi, Jiyun Roh, Yearim Jung, Yoojeong Ha, Suhjin Yang, Taesun Park

**Affiliations:** Department of Food and Nutrition, BK21 FOUR, Yonsei University, 50 Yonsei-ro, Seodaemun-gu, Seoul 120-749, Republic of Korea; wesuk42@naver.com (W.K.); vin1411@naver.com (D.C.); y20311@naver.com (J.R.); ukplay234@naver.com (Y.J.); hyj4313@naver.com (Y.H.); ysj765@naver.com (S.Y.)

**Keywords:** glucocorticoid, glucocorticoid receptor, cAMP, ERK, collagen

## Abstract

Glucocorticoid receptors (GRs) play a pivotal role in the stress response of the body, but overactivation can disrupt normal physiological functions. This study explores the role of cyclic adenosine monophosphate (cAMP) in GR activation and the associated mechanisms. We initially used the human embryonic kidney 293 cell line (HEK293) and found that cAMP enhancement, using forskolin and 3-isobutyl-1-methylxanthine (IBMX), did not alter glucocorticoid signaling under normal conditions, as evidenced by glucocorticoid response element (GRE) activity and the translocation of GR. However, in stressful conditions induced by dexamethasone, a synthetic glucocorticoid, cAMP was found to lessen glucocorticoid signaling within a short time frame but amplify it over an extended period in HEK293 cells. Bioinformatic analysis revealed that cAMP upregulation triggers the extracellular signal-regulated kinase (ERK) pathway, which influences GR translocation and ultimately regulates its activity. This stress-modulating function of cAMP was also investigated in the Hs68 dermal fibroblast line, known for its susceptibility to glucocorticoids. We found that cAMP enhancement via forskolin reduces GRE activity and reverses collagen loss in Hs68 cells exposed to dexamethasone. These findings underline the context-specific role of cAMP signaling in managing glucocorticoid signaling and its potential therapeutic application in treating stress-related pathological conditions like skin aging characterized by collagen reduction.

## 1. Introduction

Glucocorticoids are hormones synthesized endogenously in the adrenal cortex, primarily in response to various physical and psychological stressors, and are subsequently released into the bloodstream. These hormones can also be produced in other tissues and organs, such as the skin. Typically, elevated glucocorticoid levels return to normal due to a process of negative feedback. However, under conditions of chronic stress, abnormally high concentrations of glucocorticoids often persist. In addition to endogenous synthesis, glucocorticoids can also be introduced directly into the human body via potent topical agents. These agents are often used to treat inflammatory skin diseases, including atopic dermatitis. These glucocorticoids interact with specific glucocorticoid receptors located within the cytoplasm of target cells [1,2,3,4,5]. Once the glucocorticoids bind to their receptors (glucocorticoid receptors; GRs), it results in the activation and subsequent translocation of the receptor from the cytoplasm to the nucleus. In the nuclear environment, the activated receptor binds to specific genomic sequences known as glucocorticoid response elements (GREs). This binding event initiates the transcription of a range of target genes, which are instrumental in facilitating the complex adaptation mechanisms of the body to stress [6,7,8]. Despite their beneficial role, an excess of these hormones can lead to undesirable outcomes by modifying the standard physiological processes, particularly in the skin. For instance, increased levels of glucocorticoids can induce a decrease in collagen synthesis in dermal fibroblasts, potentially causing skin aging [9,10,11]. Therefore, gaining a comprehensive understanding of how to modulate glucocorticoid signaling becomes imperative in order to control and mitigate these undesirable side effects, particularly in contexts where their levels are abnormally high.

Cyclic adenosine monophosphate (cAMP) is a ubiquitous second messenger in eukaryotic cells that plays an instrumental role in the conversion of extracellular signals into intracellular responses [12,13]. This process begins with external stimuli, which stimulate the synthesis of cAMP through adenylate cyclase (ADCY). Subsequently, cAMP activates protein kinase A (PKA), a key player in the transduction pathway, which affects a wide array of responses through the phosphorylation of specific target proteins [13,14,15]. In addition, the cellular level of cAMP is tightly regulated by phosphodiesterases (PDEs), which hydrolyze cAMP to AMP, thereby modulating the intensity and duration of the second messenger signal. This dynamic interplay of synthesis, activation, and degradation enables cAMP to orchestrate numerous cellular events [16,17]. As such, the overarching importance of cAMP extends beyond its role as a second messenger; it serves as a central coordinator of myriad cellular events, facilitating fine-tuning of numerous pathways and ensuring the maintenance of cellular homeostasis [18,19,20].

Traditionally, glucocorticoid and cAMP signaling have been recognized as separate pathways mediating intracellular responses to external stimuli. However, accumulating studies suggest a more nuanced relationship between these two. For instance, glucocorticoids have been found to interact with the noradrenergic signaling pathway, thereby activating cAMP and modulating the beta-adrenoceptor-cAMP system, which in turn influences memory consolidation [21]. Additionally, glucocorticoids have been shown to amplify cAMP-dependent bronchodilation by directly increasing cAMP levels [22]. Another study posits that glucocorticoids improve the ability of hepatocytes to accumulate cAMP through protein synthesis-dependent processes; this involves, at least partially, a reduction in cAMP degradation [23]. In contrast, the influence of the cAMP pathway on glucocorticoid signaling has not been extensively explored. In this study, we aim to determine the effects of cAMP activation on glucocorticoid signaling activity and explore the mechanisms by which it does so.

## 2. Results

### 2.1. cAMP Does Not Affect Glucocorticoid Signaling under Non-Stressful Conditions

The objective of the current study was to elucidate the role of cAMP in the stress response, primarily characterized by the activation of glucocorticoid signaling. To do this, we used forskolin and 3-isobutyl-1-methylxanthine (IBMX) as cAMP activators to enhance cAMP levels. The study utilized human embryonic kidney 293 cells (HEK293), which served as a model system due to their high transfection efficiency. Before exploring the effects of heightened cAMP on the stress response, we aimed to verify the efficacy of forskolin and IBMX in activating the cAMP pathway and optimize the concentration and duration of forskolin and IBMX treatments to effectively raise cAMP levels.

**Figure 1:**
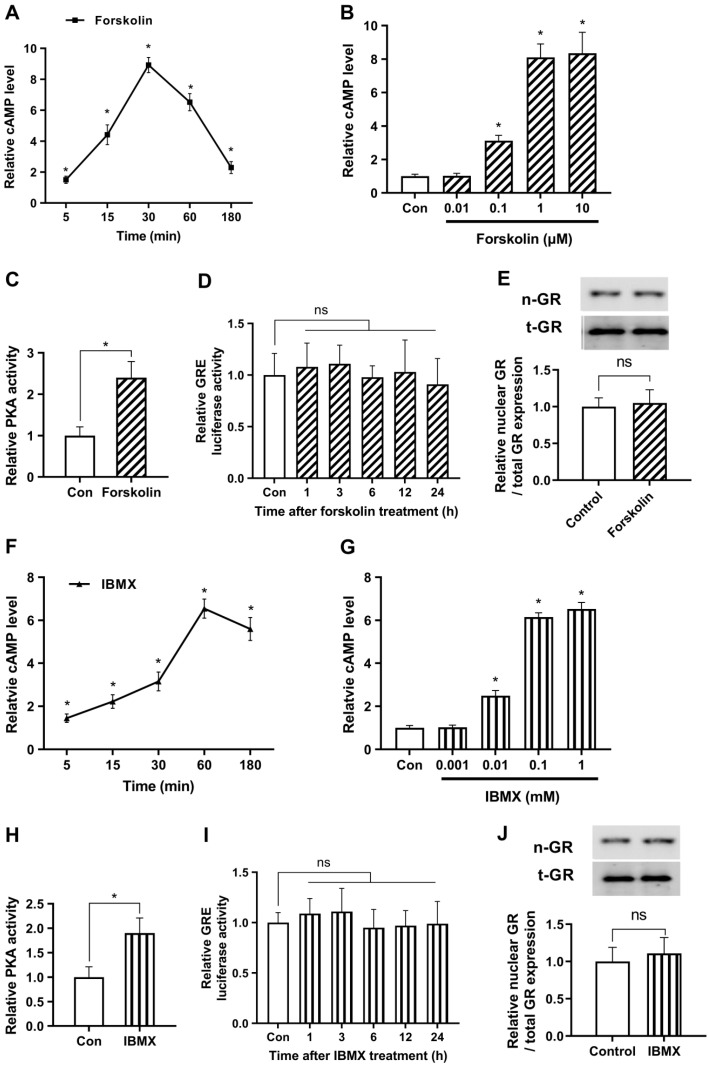
cAMP does not affect glucocorticoid signaling under non-stressful conditions. The study utilized human embryonic kidney 293 cells (HEK293) as a model system for easy transfection. (**A**) Cells were treated with 1 μM forskolin and relative cAMP levels were quantified over time by enzyme-linked immunosorbent assay (ELISA). (**B**) To optimize the concentration of forskolin, cells were exposed to various concentrations of forskolin, and relative cAMP levels were verified after 30 min using ELISA. (**C**) To identify the impact of increased levels of forskolin on downstream mechanisms of cAMP, protein kinase A (PKA) activity was assessed. Cells were subjected to a 30 min treatment with 1 μM forskolin, and the levels of PKA were quantified using an ELISA assay. (**D**) The glucocorticoid response element (GRE) plasmid was transfected into the cells with the Renilla luciferase reporter plasmid vector (pRL-TK) as an internal control for transfection efficiency. After 24 h cells were treated with 1 μM forskolin and the luciferase assay was conducted over time. (**E**) Cells were cultured with either 1 μM forskolin or vehicle for 24 h, and nuclear translocation of GR was measured by Western blot (n-GR, nuclear GR; t-GR, total GR). (**F**) To determine the optimal duration of 3-isobutyl-1-methylxanthine (IBMX) treatment, cells were treated with 0.1 mM IBMX and relative cAMP levels were measured over time using ELISA. (**G**) Cells were exposed to different concentrations of IBMX and relative cAMP levels were measured after 60 min using ELISA. (**H**) To assess the impact of increased levels of IBMX on downstream mechanisms of cAMP, PKA activity was evaluated. Cells were treated with 0.1 mM IBMX for 60 min and the levels of PKA were quantified using an ELISA assay. (**I**) The Cells were transfected with a plasmid containing the GRE and a pRL-TK vector. After 24 h, the cells were treated with IBMX, and the response was monitored over time using a luciferase assay. (**J**) The cells were grown for 24 h with 0.1 mM IBMX or vehicle and the nuclear translocation of GR was determined. The results are expressed as mean ± standard error of the mean (SEM) of three biological replicates (* *p* < 0.05; ns, not significant).

We sought to examine the time-dependent variations in cAMP production after treatment with 1 μM of forskolin, as a representative concentration. As depicted in Figure 1A, we observed a gradual increase in cAMP levels over time, peaking at 30 min post-treatment (276.5 ± 15.2 pmol/mg protein), and then subsequently declining. Expanding on these findings, we further evaluated the dose-dependent augmentation of cAMP levels in response to forskolin treatment for 30 min. Our examination revealed a steady increase in cAMP levels that plateaued at concentrations exceeding 1 μM (Figure 1B). Based on these observations, we elected to employ a concentration of 1 μM for subsequent experiments. To investigate the impact of forskolin on the downstream mechanisms of cAMP, we assessed the activity of PKA, a renowned effector of cAMP. As shown in Figure 1C, PKA activity was markedly increased following forskolin treatment, indicating considerable upregulation of cAMP signaling.

To evaluate the effect of forskolin on glucocorticoid signaling, we conducted a GRE luciferase assay. The results indicated that the forskolin-induced cAMP surge did not induce a significant change in glucocorticoid signaling (Figure 1D). Subsequently, we evaluated the nuclear translocation of GR, which is considered an alternative marker for GR activity, using Western blotting. We first verified that the nuclear fraction was properly extracted in the present experimental conditions (Figure S1). Next, we demonstrated that the nuclear expression levels of GR remained unaltered by forskolin (Figure 1E). These findings provide evidence that forskolin does not affect glucocorticoid signaling under non-stressful conditions.

To supplement the previously mentioned strategies, we optimized the concentration and duration of IBMX treatment to elevate cAMP levels. First, we investigated the temporal fluctuations of cAMP synthesis in response to exposure to 0.1 mM IBMX as a representative concentration. Our results indicate a progressive rise in cAMP levels, with a maximum observed around the 60-min time point after treatment (202.7 ± 13.9 pmol/mg protein), subsequently followed by a decline (Figure 1F). Furthermore, we found that the observed elevation in cAMP levels reached a plateau at concentrations exceeding 0.1 mM (Figure 1G). We measured PKA activity to examine the effects of elevated IBMX levels on the mechanisms downstream of cAMP. PKA activity significantly increased after 0.1 mM of IBMX treatment, as seen in Figure 1H. However, no significant alterations were detected in the glucocorticoid pathway as determined by a GRE luciferase assay or in the protein expression levels of nuclear GR following IBMX treatment (Figure 1I,J). The findings of our study were that alterations in cAMP levels, whether through enhanced synthesis or decreased degradation, do not influence glucocorticoid signaling under non-stressful conditions.

### 2.2. cAMP Alleviates Glucocorticoid Signaling under Stressful Conditions in a Short Period

**Figure 2:**
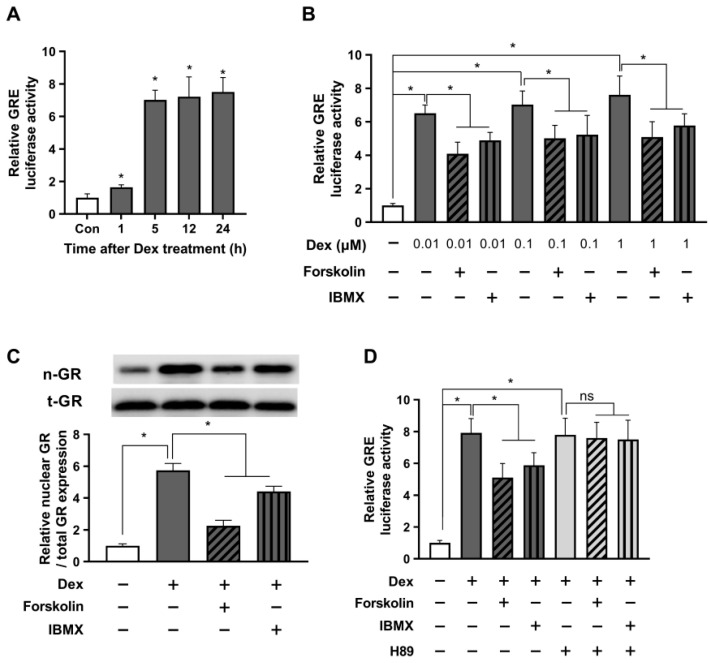
cAMP alleviates glucocorticoid signaling under stressful conditions in a short period. The GRE plasmid was transfected into HEK293 cells with the pRL-TK vector as an internal control. (**A**) After 24 h, the cells were incubated with either 0.1 μM dexamethasone or a parallel vehicle for 1, 5, 12, and 24 h. Subsequently, the cells were lysed for a GRE luciferase assay. (**B**) 24 h post-transfection, the cells were incubated with dexamethasone at indicated concentrations, 1 µM forskolin, or 0.1 mM IBMX for 5 h. The cells were harvested, and the GRE luciferase activity was measured. (**C**) The cells were exposed to 1 µM dexamethasone with 1 µM forskolin or 0.1 mM IBMX for 5 h. The nuclear translocation of GR was assessed using Western blot. (**D**) Transfected cells were pretreated with H89 (10 µM) for 30 min. Subsequently, cells were exposed to 1 µM dexamethasone with either 1 µM forskolin or 0.1 mM IBMX. The cells were then incubated for 5 h and lysed for the GRE luciferase assay. The data are expressed as mean ± SEM of three biological replicates (* *p* < 0.05; ns, not significant).

Recognizing the possibility that cAMP action could vary between stressful and non-stressful conditions, we sought to investigate the role of cAMP in modulating the glucocorticoid signaling under stressful conditions induced by dexamethasone, a synthetic glucocorticoid in HEK293. To validate this hypothesis, we initiated experiments to examine the time-dependent dynamic patterns of glucocorticoid signaling activation induced by 0.1 μM dexamethasone and monitored GRE activity over time using a luciferase assay. The data revealed a significant increase in GRE activity 5 h post-treatment, which remained elevated until 24 h (Figure 2A). Based on these findings, we first selected a 5 h treatment duration of dexamethasone treatment for subsequent experiments. To investigate the impact of varying concentrations of dexamethasone on GRE activity, we conducted a GRE luciferase assay and simultaneously examined the influence of forskolin and IBMX on modulating GRE activity at each concentration. The results showed a concentration-dependent increase in GRE activity in response to dexamethasone, with a saturated increase observed at 1 μM. Meanwhile, treatment with forskolin and IBMX notably reduced GRE activity (Figure 2B).

Subsequently, we assessed the protein expression of nuclear GR to validate the findings of the previous experiment. As presented in Figure 2C, we observed a significant upregulation of nuclear GR protein levels following treatment with 1 μM dexamethasone, indicating a substantial enhancement of the glucocorticoid receptor activity. In contrast, a decline in nuclear GR expression was observed after treatment with forskolin or IBMX. Given the potential for forskolin and IBMX to exert GRE-reducing effects through off-target mechanisms other than those involving cAMP, we employed the PKA inhibitor H89 to block PKA downstream of cAMP. Our findings showed that in the presence of H89, the GRE-reducing efficiency of forskolin and IBMX was completely nullified in response to the dexamethasone-induced upregulation in GRE activity (Figure 2D). These results provide compelling evidence that the stress-alleviating effects of forskolin and IBMX are indeed mediated through the cAMP signaling pathway.

### 2.3. Prolonged Activation with cAMP Enhances Glucocorticoid Signaling

We have confirmed the inhibitory effect of cAMP on glucocorticoid signaling in the short term (5 h after treatment). Next, we treated HEK293 cells with dexamethasone in conjunction with cAMP enhancers, forskolin, or IBMX, and monitored GRE luciferase activity after 12 and 24 h of incubation to investigate the relatively long-term effects on glucocorticoid receptor signaling.

**Figure 3:**
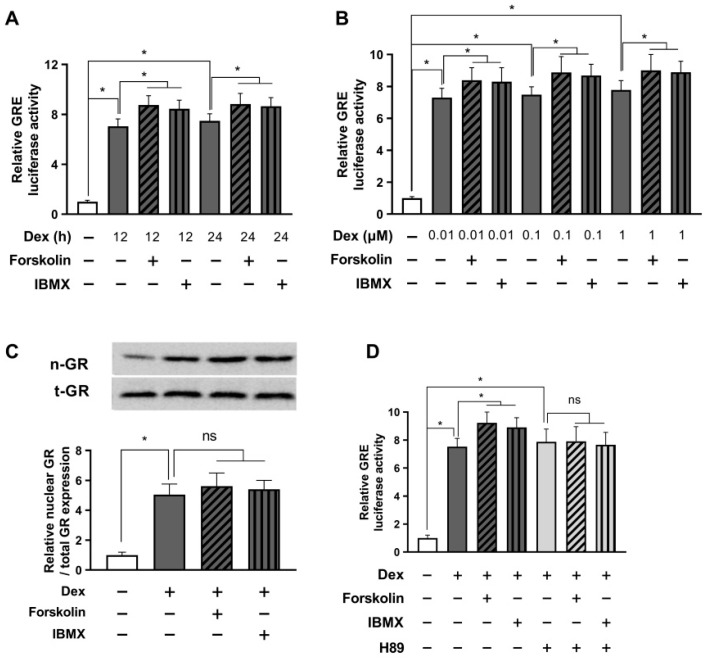
Prolonged activation with cAMP enhances glucocorticoid signaling. HEK293 cells were co-transfected with the GRE plasmid and the pRL-TK vector for an internal control. (**A**) After 24 h of transfection, cells were treated with 1 µM dexamethasone and either 1 µM forskolin or 0.1 mM IBMX for 12 or 24 h. The GRE luciferase assay was conducted after cell harvest. (**B**) Cells were treated with indicated concentrations of dexamethasone and either 1 µM forskolin or 0.1 mM IBMX for 24 h. Subsequently, GRE luciferase activity was measured after cell lysis. (**C**) Cells were treated with 1 µM dexamethasone and either 1 µM forskolin or 0.1 mM IBMX for 24 h. Afterward, the relative nuclear/total GR expression was evaluated by Western blot. (**D**) Transfected cells were pretreated with H89 (10 µM) for 30 min and then exposed to 1 µM dexamethasone and either 1 µM forskolin or 0.1 mM IBMX. The GRE luciferase assay was performed after cell incubation for 24 h. The results are reported as mean ± SEM of three biological replicates (* *p* < 0.05; ns, not significant).

The data revealed that after 12 h of combined treatment with dexamethasone and cAMP enhancers, GRE activity returned to baseline levels, showing a slight, non-significant increase compared to the group treated with dexamethasone alone. Interestingly, 24 h after administering dexamethasone and cAMP enhancers, we observed a significant rise in GRE activity, compared to dexamethasone treatment alone (Figure 3A). We opted for a 24 h treatment duration and ran the GRE luciferase assay to evaluate the effects of different dexamethasone concentrations on GRE activity, and the influence of forskolin and IBMX on GRE modulation. Impressively, our data demonstrated that cAMP enhancers significantly elevated the GRE activity induced by each concentration of dexamethasone (Figure 3B).

Next, we performed a Western blot to assess nuclear GR translocation. As seen in Figure 3C, treatment with forskolin or IBMX did not affect the nuclear GR translocation, suggesting that an increase in GRE due to long-term treatment with forskolin or IBMX does not involve the GR translocation mechanism. To investigate whether the enhanced stress response induced by forskolin or IBMX treatment operates via a cAMP mechanism, we carried out a GRE luciferase assay using the PKA inhibitor H89. The results showed that in the presence of H89, the potential of forskolin and IBMX to increase GRE activity in response to dexamethasone-induced stimulation was completely nullified (Figure 3D). These findings strongly support the idea that prolonged activation with cAMP can enhance glucocorticoid signaling, which is contrary to the results observed in our earlier short-term experiments and appear to be a typical negative feedback phenomenon of glucocorticoid receptor signaling.

### 2.4. The ERK Pathway Is Involved in the Stress-Relieving Effects of cAMP

**Figure 4:**
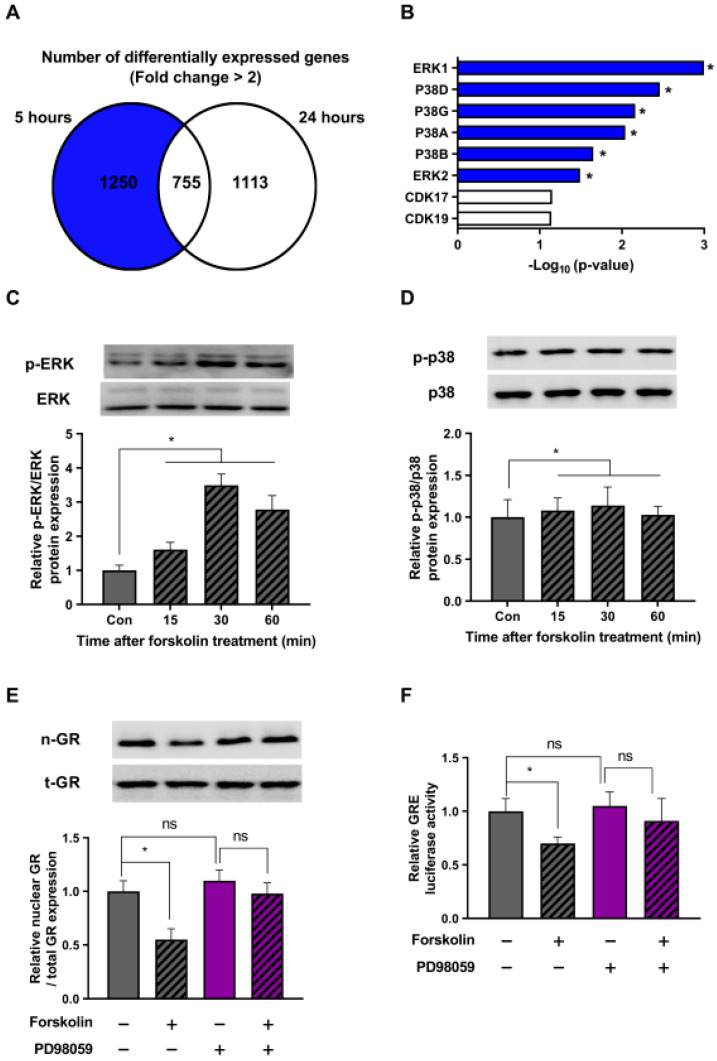
The ERK pathway is involved in the stress-relieving effects of cAMP. (**A**) HEK293 cells were treated with 1 μM dexamethasone and 1 μM forskolin for 5 or 24 h. Following the treatment, RNA sequencing was performed using next-generation sequencing (NGS) technology. A Venn diagram represents the number of genes showing an increase in mRNA expression of more than two-fold when treated with dexamethasone and forskolin, in comparison to the control treated with dexamethasone alone. (**B**) A comprehensive bioinformatics analysis using Enrichr was performed on a set of 1250 previously identified genes. (**C**,**D**) HEK293 cells exposed to 1 μM dexamethasone were treated with 1 μM forskolin for different durations. Subsequently, Western blot analysis was performed using phospho-specific antibodies against ERK and p-38. (**E**) HEK293 cells in the presence of dexamethasone (1 μM) were pretreated with 50 μM PD98059 and exposed to 1 μM forskolin for 5 h. The subsequent translocation of GR to the nucleus was analyzed by Western blot. (**F**) HEK293 cells were transfected with the GRE plasmid and the pRL-TK vector as an internal control. After 24 h of transfection, the cells were pretreated with 50 μM PD98059 (indicated in purple) and incubated with 1 μM forskolin in the presence of dexamethasone for 5 h. Subsequently, a GRE luciferase assay was performed after incubation. Panels (**A**,**B**) displays the results from a single replicate, whereas Panels (**C**–**F**) presents the mean ± SEM of three biological replicates (* *p* < 0.05; ns, not significant).

The main focus of this study was to explore the mechanisms by which cAMP regulates the stress response, specifically in a downward direction. For this, we conducted a bioinformatics analysis using Enrichr on a subset of 1250 genes compared to the dexamethasone-only control group, excluding the 755 genes at the intersection that showed a more than a two-fold increase (Figure 4A). Taking into account the role of cAMP–PKA in various protein phosphorylations, our hypothesis centered around the potential involvement of kinase proteins. Bioinformatics analysis provided compelling evidence suggesting the likely activation of ERK and P38 proteins following forskolin treatment (Figure 4B).

To validate our bioinformatics findings, we checked the phosphorylation state of the ERK and p38 proteins in response to forskolin treatment in the presence of dexamethasone at various time intervals. Our results showed that forskolin treatment triggered the activation of the ERK protein, with peak phosphorylation observed at 30 min (Figure 4C). On the contrary, we detected no increase in the phosphorylation of the p38 protein at any time point after forskolin treatment (Figure 4D). These findings suggest that cAMP plays a crucial role as a regulator of the ERK signaling pathway in stressed cells.

We further investigated whether the cAMP-induced increase in ERK phosphorylation could indeed affect GR translocation. To explore this, we conducted additional experiments using forskolin and an ERK inhibitor (PD98059) and evaluated the cellular distribution of GR. Our results showed that forskolin treatment hindered the intranuclear translocation of GR, and this effect was reversed when the ERK inhibitor was present (Figure 4E). Additionally, as measured by the luciferase assay, our results in Figure 4F demonstrate that the ability of forskolin to reduce GRE activity was nullified with the addition of an ERK inhibitor. Taken together, these findings suggest that the ERK pathway is critically involved in the stress-relieving effects of cAMP.

### 2.5. cAMP-Mediated Stress Reduction Improves Collagen Levels in Human Dermal Fibroblasts

We have demonstrated the impact of cAMP on glucocorticoid signaling in HEK293 cells, prompting our interest in exploring its reproducibility in other human cell lines. For this purpose, we utilized the human dermal fibroblast cell line (Hs68), which is known to be vulnerable to stress and downregulate collagen levels. Our objective was to see whether modulating glucocorticoid signaling through cAMP could control stress-induced collagen reduction in human dermal fibroblasts.

**Figure 5:**
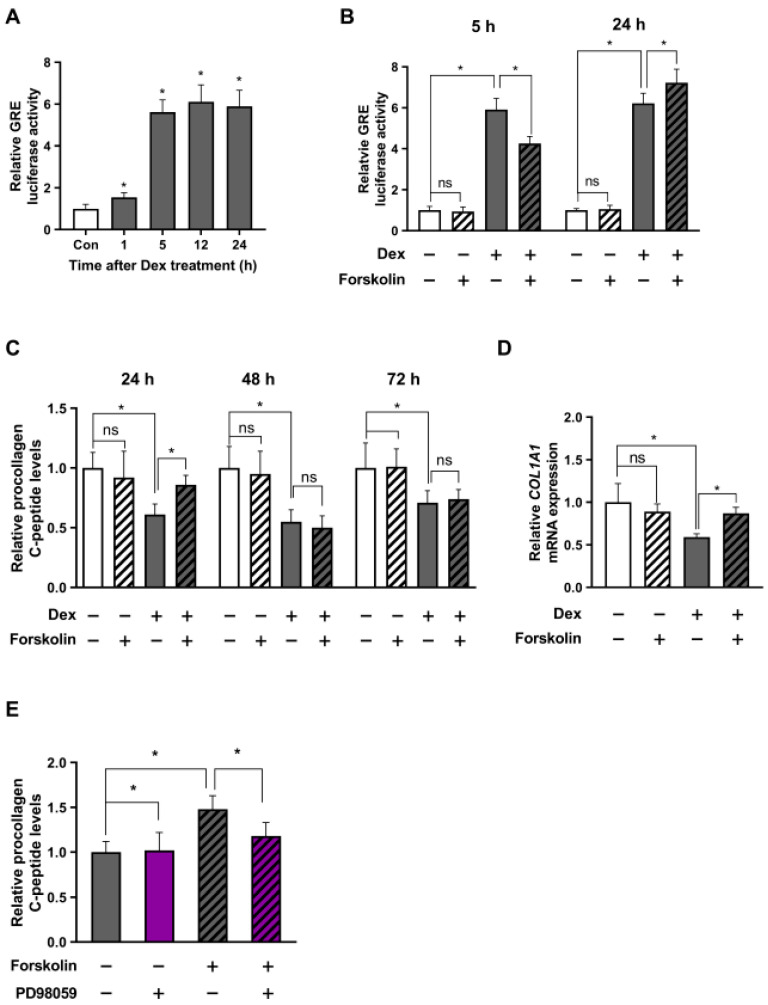
cAMP-mediated stress reduction improves collagen levels in human dermal fibroblasts. Human dermal fibroblast Hs68 cells were co-transfected with the GRE plasmid and the pRL-TK vector for normalization control. (**A**) After 24 h, the cells were treated with 1 μM dexamethasone for 1, 5, 12, and 24 h. The cells were then harvested, and a GRE luciferase assay was conducted. (**B**) Transfected cells were treated with dexamethasone alone (1 μM), forskolin alone (1 μM), or a combination of dexamethasone and forskolin. After incubation for 5 or 24 h, the cells were lysed, and a GRE luciferase assay was performed. (**C**) Hs68 cells were exposed to dexamethasone alone (1 μM), forskolin alone (1 μM), or dexamethasone and forskolin together for 24, 48, or 72 h. The culture supernatants of dermal fibroblasts were collected daily to measure the content of procollagen type I C-peptide using ELISA. (**D**) Additionally, 24 h after treatment, the gene expression levels of collagen type I α1 chain (COL1A1) in the cells were quantitated using qRT-PCR. (**E**) Hs68 cells exposed to dexamethasone were initially exposed to μM PD98059 (50 μM; indicated in purple) and treated with forskolin (1 μM) for 24 h. Subsequently, the content of procollagen type I C-peptide was measured using ELISA after 24 h. The values are presented as the mean ± SEM of three biological replicates (* *p* < 0.05; ns, not significant).

We examined the activity of GRE in the Hs68 cell line following dexamethasone treatment, with a focus on both short- and long-term stress effects. The data showed a significant increase in GRE activity at the 5 h mark after treatment, which remained high for 24 h (Figure 5A). As depicted in Figure 5B, GRE activity in the presence of dexamethasone decreased after 5 h of forskolin treatment (short-term) but increased after 24 h of treatment (long-term).

**Figure 6:**
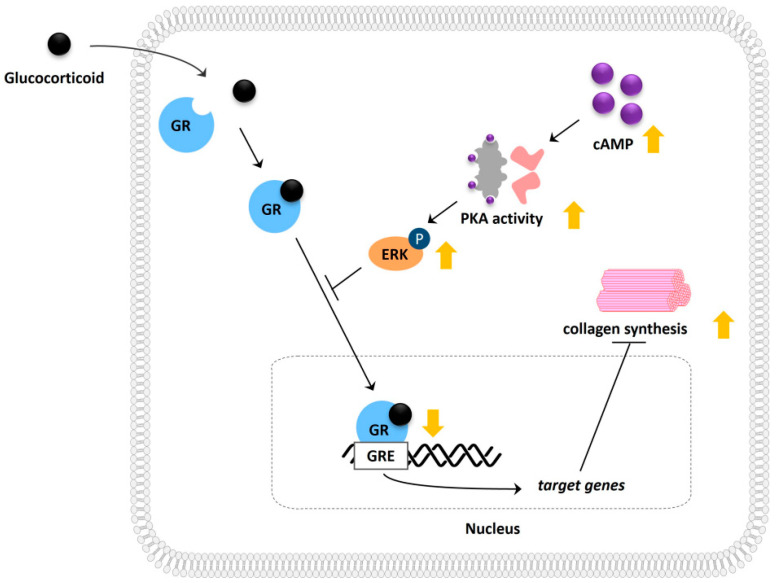
Schematic illustration depicting the molecular mechanism involved in cAMP-mediated modulation of glucocorticoid signaling and its potential effect on collagen synthesis.

Next, we measured procollagen levels in the supernatants of Hs68 cells after short-term (24 h) and long-term (48 h, 72 h) treatment with dexamethasone and forskolin. We noted a noticeable decrease in procollagen levels under the stress conditions induced by dexamethasone. Treatment with forskolin alone did not significantly affect procollagen levels at any time point. In particular, short-term treatment with forskolin in conjunction with dexamethasone restored the decreased collagen levels, while long-term treatment did not significantly alter the collagen levels (Figure 5C). To further validate this significant observation, we examined mRNA expression using qPCR and confirmed that short-term forskolin treatment reduced the gene expression levels of collagen type I α1 chain (COL1A1) in the presence of dexamethasone (Figure 5D). In addition, we found that the cAMP-induced increase in collagen under stress was partially reduced in the presence of the ERK inhibitor PD98059 (Figure 5E). These findings suggest the regulatory role of cAMP in stress-induced collagen reduction, with a partial contribution from ERK mechanisms (Figure 6).

## 3. Discussion

The therapeutic potential of cAMP for a variety of skin-related conditions is widely recognized. Numerous studies suggest that elevated cAMP levels may effectively control inflammation and itchiness, symptoms characteristic of atopic dermatitis, a chronic inflammatory skin condition [24,25,26]. Furthermore, some research indicates that agents enhancing cAMP levels could inhibit the growth of melanoma cells, thus providing a promising strategy for melanoma treatment [27,28,29,30]. In our study, we focused on type 1 collagen, a crucial protein that imparts strength and elasticity to the skin. It is well-documented that the production of this protein decreases not only due to intrinsic factors but also to extrinsic factors such as exposure to ultraviolet light and glucocorticoids, resulting in skin aging characterized by wrinkles and sagging skin [31,32,33]. We harnessed the potential of cAMP to counteract glucocorticoid activity, mitigating the decrease in collagen production in dermal fibroblasts caused by stress. Interestingly, the initial reduction in stress facilitated by cAMP was sufficient to reverse the stress-induced loss of collagen. However, despite additional increases in stress over the long term, we did not observe additional collagen loss. This suggests that a certain degree of stress does not lead to further degradation of collagen.

The primary target of cAMP within cells is PKA, which plays a pivotal role in the phosphorylation of various proteins and consequently regulates various cellular pathways [34,35,36]. The impact of the cAMP–PKA axis on ERK phosphorylation is one of the most complex issues in cAMP-mediated protein kinase research. This issue is highly context-specific and depends on various factors, including the presence of stimuli for cell growth and cell types [37]. It has long been appreciated that several growth factors, including insulin, vascular endothelial growth factor, and fibroblast growth factor, strongly activate ERK signaling. This activation can be inhibited by cAMP, thus exerting a suppressive effect on the ERK pathway in several cell types, such as adipocytes and hepatocytes [38,39,40]. Conversely, under normal conditions, cAMP has been reported to stimulate ERK signaling in several cell types, including granulosa cells and kidney cells, predominantly involving changes in PKA [41,42,43]. In our study, we discovered that an increase in cAMP levels, without any co-treatment with specific growth factors, enhanced the ERK pathway in a dexamethasone-exposed human kidney cell line. We further confirmed the stress-reducing effects of this elevated ERK activity in a dexamethasone-induced dermal fibroblast line. However, it remains unclear whether conditions containing certain growth factors or those involving other cell types would respond in a similar way. Further research is required to understand the pattern of alteration of ERK proteins induced by cAMP in various contexts.

In order to assess the role of PKA in the regulation of GR induced by cAMP, we performed a PKA activity assay and then investigated whether the effect of a cAMP enhancer was eliminated in the presence of a PKA inhibitor, H89. However, it is important to note that although H89 is commonly used to inhibit PKA in various studies, it may also affect other kinases, such as p70S6 kinase and Rho-associated protein kinase. Therefore, we acknowledge that our methodology has certain limitations, particularly due to the relatively high dose of H89 (10 µM) used in our study, which could potentially influence other cellular processes and introduce some degree of uncertainty into our results [44,45]. For future research that investigates the mechanism in detail, it would be beneficial to employ other PKA inhibitors as well, such as Rp-cAMP, to confirm the involvement of PKA and provide a more specific effect. Alternatively, a highly selective PKA siRNA knockdown method could be utilized to ensure the specificity of PKA inhibition and distinguish the exact role of PKA.

Numerous studies, including our own previous work, have reported that cAMP itself has the ability to control collagen production through a variety of mechanisms independent of stress; however, consensus on the direction of regulation has not yet been reached. Some studies propose that an increase in cAMP levels could potentially promote the production of type 1 collagen [46,47,48], while others have shown that an increase in cAMP may, in fact, suppress this process [49,50,51]. The exact reason for this discrepancy remains largely unclear, but the extent of the increase in cAMP has been suggested as a potential determinant of collagen regulation. One study demonstrated that a slight increase in cAMP (about 1.5-fold of the control) stimulates collagen 1 synthesis, but dramatic increases in cAMP (around 150-fold of control) inhibit collagen 1 synthesis [52]. Given these parameters, the rise in cAMP levels induced by the cAMP enhancer in our study is relatively mild and seems to have no significant impact on the decline in collagen production. We speculated that when aiming to restore stress-induced collagen, it would be important to use a moderate cAMP-elevating treatment method, which is sufficient to restore stress-induced collagen but does not inhibit collagen production via an independent pathway through excessive cAMP elevation.

The intervention of glucocorticoid receptor signaling has been extensively studied at three levels: (1) Specific molecules, such as ionone, structurally attach to the GR, thereby competitively interfering with the binding of glucocorticoids [32,53,54]. (2) Protein kinases modulate the phosphorylation of the GR, regulating its activity, including the regulation of its translocation into the nucleus [55,56,57]. (3) Several transcription factors, such as nuclear factor kappa B and activator protein 1, compete with GR for DNA binding in the nucleus, consequently reducing GRE activity [58,59,60]. In this study, our primary focus was on kinases, based on the understanding that the cAMP–PKA pathway can regulate various kinase proteins. We elucidated that cAMP–PKA signaling is capable of activating ERK, with ERK1 and ERK2 being the most extensively studied. It is well-known that, once activated, ERK1/2 can phosphorylate a range of downstream target proteins, including transcription factors, which ultimately influence gene expression and, consequently, cellular function [61,62]. Although the exact mechanism remains unclear, several studies have shown that ERK may inhibit the translocation of GR, potentially involving the phosphorylation regulation at multiple serine sites [55,56,57,63]. In line with these findings, we demonstrated that ERK activation by cAMP effectively inhibits the translocation of GR to the nucleus, resulting in reduced GRE activation.

In this study, we demonstrated that the promotion of the cAMP mechanism can modulate glucocorticoid receptor signaling. Furthermore, the cAMP pathway significantly restored the dexamethasone-induced decrease in collagen in dermal fibroblasts. These findings highlight the potential of cAMP-elevating agents, at least for topical application. However, when considering systemic applications, the matter is not only about reducing the stress response in the target cell but also about inducing changes in the stress hormones themselves. Indeed, several studies provide evidence that cAMP-elevating compounds, such as forskolin, can activate PKA, which in turn stimulates the adrenal glands, primarily responsible for the generation of glucocorticoids, to produce more cortisol [64,65]. Therefore, in the case of systemic applications, such as dietary intake of cAMP activators, other factors like effects on glucocorticoid production should be considered in an integrated manner. In addition, our in vitro experimental model, with a duration of merely a few days, presents difficulties in extrapolating results to chronic stress disorders that evolve over significantly extended periods in human subjects. This presents a challenge for drawing direct comparisons between our findings and the complex realities of chronic stress pathologies in humans. Consequently, it is crucial to interpret our findings within the limitations of the model system used, and to exercise caution when generalizing these results to human chronic stress conditions. These considerations highlight the need for further exploration in future in vivo studies.

The present findings suggest that regulating cAMP levels could present a potential therapeutic strategy for managing stress-related pathological conditions. Initially, we explore the potential for mitigating skin aging by reversing collagen loss. This approach might extend to other diseases directly linked to stress and chronically upregulated glucocorticoids, such as certain mental health disorders, cardiovascular disease, and metabolic syndrome. Additionally, it could apply to conditions where stress exacerbates symptoms, such as in autoimmune diseases [66,67,68,69]. Future research can be designed to test the beneficial role of cAMP in each disease where glucocorticoid signaling is implicated.

## 4. Materials and Methods

### 4.1. Cell Culture

HEK293 cells and the human skin fibroblast (Hs68) cell line were obtained from the American Type Culture Collection (Manassas, VA, USA). These were maintained in high-glucose Dulbecco’s modified Eagle’s medium (Hyclone, Logan, UT, USA) supplemented with 10% fetal bovine serum (FBS; Hyclone) and 1% penicillin/streptomycin (Gibco, Grand Island, NE, USA). To prevent mycoplasma contamination, 1% of MycoZap antibiotics (Lonza, Basel, Switzerland) were added to the culture medium during the cell culture process. The cells were incubated in a 5% CO_2_ environment at 37 °C, and the culture medium was replaced every 2–3 days. As the cell confluence reached approximately 80%, subculturing was performed to maintain optimal growth conditions. To minimize variations in cell characteristics, all experiments were conducted using cells with a passage difference of no more than three. Additionally, cells were subcultured up to a maximum of ten passages for all experiments.

### 4.2. Transient Transfection and Luciferase Assay

Both HEK293 and Hs68 cells were grown until they reached approximately 80% confluence in 24-well plates. The cells were cultured in antibiotic-free media and co-transfected with the firefly luciferase plasmid harboring the GRE (Promega, Madison, WI, USA) and the Renilla luciferase plasmid (Promega) using Lipofectamine 3000 (ThermoFisher, Waltham, MA, USA). Following 24 h of transfection, cells were treated with the designated concentrations of dexamethasone, cAMP-elevating agents such as forskolin (Sigma-Aldrich, St. Louis, MO, USA) or IBMX (Sigma-Aldrich), or both. If required, the PKA inhibitor (H89; Sigma-Aldrich) or the extracellular signal-regulated kinase (ERK) inhibitor (PD98059; MCE, Monmouth Junction, NJ, USA) was pretreated in an incubation medium for 1 h before compound treatment. Cells were further incubated for the specified time, rinsed with ice-cold phosphate-buffered saline (PBS), and then harvested in a passive lysis buffer (Promega). For luminescence measurement, the cell lysate was mixed in sequence with the firefly luciferase reagent and transferred to a GLOMAX luminometer, followed by the addition of the Renilla luciferase reagent (Dual Luciferase Reporter assay system; Promega). The resulting luminescence was quantified with firefly luciferase activity normalized to Renilla luciferase as an internal transfection control.

### 4.3. Protein Extraction and Western Blot Analysis

HEK293 cells were seeded in 6-well plates and subjected to specified concentrations and time points of dexamethasone and forskolin, either in the presence or absence of PD98059. Cell lysates were prepared using a nuclear extraction kit (Abcam, Cambridge, MA, USA) or RIPA buffer (ThermoFisher) supplemented with protease and phosphatase inhibitors (Sigma-Aldrich). The protein concentrations of the lysates were determined using the BCA Protein Assay Kit (Takara, Tokyo, Japan), following the manufacturer’s instructions. Equal amounts of protein extracts were separated on a sodium dodecyl sulfate-polyacrylamide gel electrophoresis (SDS-PAGE) and subsequently transferred to a nitrocellulose membrane (BioRad, Hercules, CA, USA). The membrane was blocked with 5% bovine serum albumin (BSA; MP Biomedicals, Santa Ana, CA, USA) in Tris-buffered saline containing 0.1% Tween 20 (TBS-T) for 1 h. Following the blocking, the membrane was probed with primary antibodies against p-ERK (1:1000; Cell Signaling, Danvers, MA, USA), ERK (1:1000; Cell Signaling), p-p38 (1:1000; Cell Signaling), p38 (1:1000; Cell Signaling), GR (1:1000; Cell Signaling), lamin B1 (1:1000; Cell Signaling), or β-tubulin (1:1000; Cell Signaling) for 16 h at 4 °C. After incubation with the primary antibodies, the membrane was washed three times with TBS-T for 5 min each, after which the corresponding secondary antibody (1:10,000; Invitrogen, Carlsbad, CA, USA) was added and incubated for an hour at 25 °C. Following three more washes with TBS-T for 5 min each, the blots were developed using WestGlow PICO PLUS chemiluminescent substrate (Biomax, Seoul, Republic of Korea) and visualized with a ChemiDoc imaging system (ATTO, Tokyo, Japan). The intensity of each band was quantified using ImageJ software version 1.53t (NIH, Bethesda, MD, USA).

### 4.4. PKA Activity Measurements

PKA activity in HEK293 cells was determined using the PKA Colorimetric Activity Kit (EIAPKA; Invitrogen), following the manufacturer’s instructions. Briefly, cells were cultured in 24-well plates and then treated with forskolin or IBMX for indicated concentrations and time points. Following the stimulation, cells were harvested, and cell lysates were prepared. Substrate phosphorylation was determined via a colorimetric assay and the absorbance was measured at 450 nm using an Infinite M200 microplate reader (Tecan, Männedorf, Switzerland). PKA activity was assessed by calculating the units of kinase activity for each protein sample, which were then normalized to the levels observed in the untreated control samples.

### 4.5. Quantitative Assessment of Procollagen Secretion and cAMP

The evaluation of procollagen and cAMP was conducted using the enzyme-linked immunosorbent assay (ELISA). The secreted procollagen in the medium was quantified using a Procollagen Type I C-peptide ELISA kit (Takara, Shiga, Japan), following the protocols provided by the manufacturer. Briefly, cells were initially seeded with a density of 75,000 cells per well in a 24-well plate and cultured for the indicated times. The acquired medium was centrifuged to remove debris, after which the absorbance of type 1 procollagen was determined at 590 nm using an Infinite M200 microplate reader. The procollagen 1 secretion results were then adjusted to the total cell protein, determined by the Bradford method using detergent-compatible protein assay reagents (The Quick Start Bradford protein assay; BioRad).

For the evaluation of cAMP, after media removal, the cells were washed with phosphate-buffered saline (PBS) and exposed to 0.1 M hydrochloric acid for 5 min. The cAMP levels in the acquired lysates were then assessed using a cAMP ELISA kit (Enzo Life Sciences, Farmingdale, NY, USA), in accordance with the manufacturer’s protocols. In brief, the lysates were neutralized, followed by the addition of the cAMP conjugate to the IgG-coated microplate binding sites to compete with cAMP. The unbound cAMP was subsequently removed with three PBS washes. Thereafter, the substrate was precisely added to each well to ascertain the enzyme’s bound activity. After reaction termination, the relative optical density at 450 nm was estimated using a microplate reader. Finally, the cAMP concentration was adjusted to the total intracellular protein concentration, measured by the Bradford method using compatible protein assay reagents (The Quick Start Bradford protein assay; BioRad).

### 4.6. RNA Extraction and Real-Time PCR (RT-PCR)

Total RNA was isolated from HEK293 and Hs68 cells using TRIzol reagent (Invitrogen), followed by chloroform extraction and isopropanol precipitation. The RNA was eluted in nuclease-free water, quantified using a NanoDrop spectrophotometer (Tecan), and reverse transcription was performed using a PrimeScript RT reagent kit with gDNA Eraser (Takara) as per the manufacturer’s protocol. RT-PCR was carried out with a final volume of 20 µL containing 10 µL of the TB Green Premix Ex Taq II (Takara), primers at a concentration of 0.3 µM each, and 10 ng of complementary DNA. The thermal cycling protocol included an initial denaturation at 95 °C for 30 s, followed by 35 cycles of denaturation at 95 °C for 5 s, and annealing and extension at 60 °C for 30 s. The data were analyzed by the 2^–∆∆Ct^ method and the expression of each gene was normalized to that of GAPDH. The primer sequences are shown in Supplementary Table S1.

### 4.7. RNA Sequencing and Bioinformatic Analysis

RNA extraction was performed as described above. The NEBNext Ultra II Directional RNA-Seq Kit (New England BioLabs, Hitchin, UK) was used in the construction of mRNA sequencing libraries. In short, mRNA was isolated from total RNA using the Poly(A) RNA Selection Kit (Lexogen, Vienna, Austria). After being purified, the mRNA underwent chemical fragmentation, resulting in short pieces that were used as templates for reverse transcriptase to create complementary DNA (cDNA). This was followed by removing the small fragments through another purification step. PCR amplification was performed after end repair, adapter ligation, and the inclusion of index codes in the specimens. The non-aligned adapters were removed twice after adapter ligation and PCR amplification. The library quantification was determined using the StepOne Real-Time PCR System (Life Technologies, Carlsbad, CA, USA), and library quality was evaluated using the Agilent 2100 bioanalyzer DNA high sensitivity kit. The libraries were sequenced using the paired-end 100 technique on an Illumina HiSeq X10 sequencing platform (San Diego, CA, USA). Gene set enrichment analysis was performed using the Enrichr tool to identify gene sets and related pathways enriched by differentially expressed genes [70]. The analysis was executed employing default parameters and referencing the Human Kinase Library 2023 database. The *p*-value was obtained based on the fold change, and a value of less than 0.05 was considered significant.

### 4.8. Statistical Analysis

Data are presented as mean ± standard error of the mean (SEM). Differences between the two groups were analyzed using SPSS software version 26.0 (SPSS, Armonk, NY, USA) by two-tailed Student’s *t*-test. The significance level was set at * *p* < 0.05.

## 5. Conclusions

In summary, this study sheds light on the role of cAMP in glucocorticoid signaling, a response that varies by context. Under non-stressful conditions, cAMP enhancement via forskolin and IBMX did not influence glucocorticoid signaling. Interestingly, this study revealed the dual effects of cAMP in stressful situations—diminishing glucocorticoid signaling within a short duration but intensifying it over a longer period. Furthermore, we discovered that increased cAMP stimulates the ERK pathway, subsequently governing GRE activity, paving the path for future exploration of the cAMP–ERK–GR signaling axis. Further supporting our findings, we demonstrated the role of cAMP in a pathophysiological context using the Hs68 dermal fibroblast cell line. In these cells, cAMP elevation successfully counteracted the deleterious effects of glucocorticoid exposure, reversing collagen loss. This research not only deepens the understanding of cAMP and glucocorticoid signaling interplay but also hints at the therapeutic potential for stress-related disorders such as skin aging.

## Data Availability

The data that support the findings of this study are available from the corresponding author upon reasonable request.

## Supplementary Materials

The supporting information can be downloaded at: https://www.mdpi.com/article/10.3390/ijms241210180/s1.
