# Supplementary material for: The Role of Cyclic Adenosine Monophosphate (cAMP) in Modulating Glucocorticoid Receptor Signaling and Its Implications on Glucocorticoid-Related Collagen Loss
Source: Int J Mol Sci. 2023 Jun 15;24(12):10180. doi: 10.3390/ijms241210180 (PMC10299417; doi:10.3390/ijms241210180)
Supplement: Supplementary file 1 [file ijms-24-10180-s001.zip › ijms-2435293-supplementary.pdf]

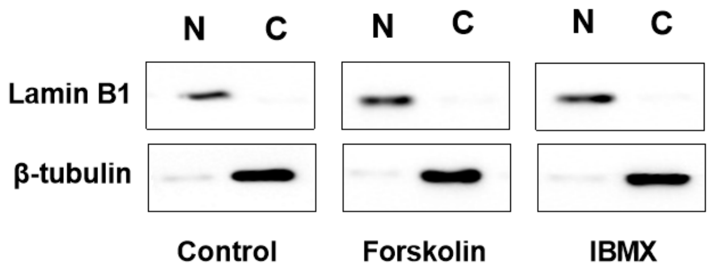

Supplementary Figure S1. Verification of nuclear fraction. Cells were cultured with either 1 μM forskolin, 0.1 mM IBMX or vehicle (control) for 24 h. Subsequently, nuclear proteins (N) and cytoplasmic proteins (C) were isolated from the cells. Western blot analysis was conducted using Lamin B1 as a nuclear marker and β-tubulin as a cytoplasmic marker to confirm the proper extraction and separation of the nuclear fraction.

Supplementary Table S1. Primer sequences for RT-qPCR.

Gene description	Sequence (5′→3′)
Collagen type Ⅰ α 1 chain (*COL1A1*)	F: ACATGTTCAGCTTTGTGGACC<br>R: TGTACGCAGGTGATTGGTGG
Glyceraldehyde-3-phosphate dehydrogenase (*GAPDH*)	F: TCTGGAAAGCTGTGGCGTGA<br>R: TACTTGGCAGGTTTCTCCAGG
